# Oral Administration of Berberine Hydrochloride Based on Chitosan/Carboxymethyl-β-Cyclodextrin Hydrogel

**DOI:** 10.3390/polym16162368

**Published:** 2024-08-21

**Authors:** Bukatuka Futila Clemence, Lin Xiao, Guang Yang

**Affiliations:** 1 Department of Biomedical Engineering, College of Life Science and Technology, Huazhong University of Science and Technology, Wuhan 430074, China; clemsbuka326@gmail.com; 2 School of Biomedical Engineering, Sun Yat-Set University, Shenzhen 518107, China

**Keywords:** berberine hydrochloride, chitosan/carboxymethyl-β-cyclodextrin, hydrogel formulation, solubility enhancement, oral drug delivery

## Abstract

In this study, a novel oral formulation of berberine hydrochloride (BBH) hydrogel was successfully synthesized through physical cross-linking using chitosan (CS) and carboxymethyl-β-cyclodextrin (CMCD). The characterization results confirmed the successful synthesis of the CS/CMCD hydrogel and the subsequent loading of BBH into this composite (CS/CMCD/BBH) was effectively accomplished. The BBH was used as a model drug and the resulting hydrogel demonstrated a sustained drug release profile. In addition to its improved solubility and sustained release characteristics, the hydrogel exhibited excellent antibacterial activity against common pathogens such as *Escherichia coli* (*E. coli*), *Staphylococcus aureus* (*S. aureus*), and *Candida albicans* (*C. albicans*). Additionally, in vitro studies indicated that the hydrogel was not cytotoxic to NIH3T3 and HaCaT cells, suggesting its safety for biomedical applications. This lack of cytotoxic effects, combined with the mechanical strength, solubility improvements, and antibacterial properties of the hydrogel, positions the CS/CMCD/BBH hydrogel as a promising candidate for the effective oral delivery of BBH. By addressing the solubility and delivery challenges of BBH, this hydrogel offers a viable solution for the oral administration of BBH, with potential applications in various biomedical fields.

## 1. Introduction

Berberine hydrochloride is a natural alkaloid with a wide range of therapeutic applications, including antimicrobial [1,2,3,4], anti-inflammatory [5], anticancer [6,7,8,9], and antidiabetic effects [10]. Despite its potential, the clinical use of BBH has been severely limited by its low solubility and stability, particularly when administered orally [11,12,13]. These challenges require the development of innovative drug delivery systems to improve their bioavailability and therapeutic efficacy [14,15]. Hydrogels have emerged as a promising solution for drug delivery, capable of improving the solubility and stability of hydrophobic drugs [16,17]. Among the different hydrogel-forming materials, chitosan and carboxymethyl-β-cyclodextrin deserve special attention [18]. Chitosan is a biocompatible polymer with excellent film-forming properties, while carboxymethyl-β-cyclodextrin is known for its ability to form inclusion complexes with hydrophobic molecules, thereby improving their solubility [19,20]. The combination of chitosan and β-cyclodextrin has been shown to be particularly effective in drug delivery systems. Chitosan cross-linked with β-cyclodextrin forms a hydrogel that acts as an excellent carrier for poorly water-soluble drugs [21,22]. β-Cyclodextrin is a cyclic oligosaccharide composed of seven glucose units [23,24], with a hydrophobic internal cavity and a hydrophilic external surface, allowing it to form inclusion complexes with hydrophobic drugs such as BBH [25,26]. β-Cyclodextrin can improve the solubility, stability, and bioavailability of poorly soluble drugs by encapsulating them in its cavity [27,28]. By combining the advantages of chitosan and β-cyclodextrin, a physically cross-linked CS/CMCD hydrogel can be developed as an effective carrier for BBH [29]. This approach avoids the need for chemical cross-linking agents, thereby preserving the biocompatibility and bioactivity of the encapsulated drug [30]. The hydrogel matrix can facilitate the sustained release of BBH, ensuring a prolonged therapeutic effect and minimizing the frequency of administration [31]. Studies have explored the application of CS/β-cyclodextrin hydrogels as drug carriers, demonstrating their potential to improve drug delivery [31,32]. For example, Hui et al. developed a BBH-loaded chitosan/β-cyclodextrin hydrogel that showed improved solubility, sustained release, and enhanced antibacterial activity [33]. Another study by Lin et al. investigated a nanocellulose/β-cyclodextrin hydrogel for the ocular delivery of BBH, which exhibited sustained drug release and improved therapeutic efficacy [34]. Mohamadi and co-authors developed a CS/CMCD-based hydrogel that significantly enhanced the antibacterial activity when loaded with silver nanoparticles [35]. Similarly, Wang et al. created a thermoresponsive CS/CMCD hydrogel for the intranasal delivery of berberine hydrochloride, resulting in better bioavailability and improved therapeutic effects [36]. Tsai et al. investigated chitosan hydrogels for the transdermal delivery of BBH and showed that the hydrogel formulation effectively improved the therapeutic outcomes in a mouse model of psoriasis. This indicating its potential as a delivery system for the transdermal administration of BBH [32,37]. Therefore, these results highlight the versatility and effectiveness of CS/CMCD hydrogels as drug delivery systems [38,39]. In this study, we developed an oral, physically cross-linked hydrogel formulation of BBH by loading it onto a CS/CMCD hydrogel matrix. The physical cross-linking interaction between the CS and CMCD led to the formation of stable complexes, exploiting the solubility-enhancing properties of CMCD as well as the biocompatibility and stability offered by chitosan. Our results demonstrate that the resulting hydrogel formulation not only significantly increases the solubility of BBH but also ensures its sustained release and improved bioavailability, thereby addressing the major limitations of free BBH in oral administration. Our results demonstrate the superior performance of this innovative formulation compared to free BBH.

## 2. Materials and Methods

### 2.1. Chemicals, Microbial Strains, and Cell Strains

Chitosan (purity > 98%),acetic acid and the 50 kDa cutoff dialysis membrane were obtained from Aladdin Industrial Biotechnology Co., Ltd. (Shanghai, China); carboxymethyl-β-cyclodextrin (purity > 98%) was purchased from Binzhou Zhiyuan Biotechnology Co., Ltd. (Binzhou, China); berberine hydrochloride (purity ≥ 98%) was obtained from Chemical Reagent Co., Ltd. (Shanghai, China)*. Escherichia coli*, *Staphylococcus aureus*, and *Candida albicans,* as well as fibroblasts or NIH3T3 cells and epithelial cells or HaCaT cells were obtained from Tongji Medical College of Huazhong University of Science and Technology.

### 2.2. Selection of Detection Wavelength

BBH was prepared according to the method of Huang et al. [25] with slight modifications. First, the wavelength was selected by accurately weighing 0.05000 g of the BBH with an analytical balance, dissolving it in a 500 mL beaker, and then diluting it with a 1 L volumetric flask to obtain a certain concentration of the BBH solution. A Thermo Fisher Scientific Multiscan-GO full-wavelength microplate reader was used to scan the spectrum of the BBH solution, and water was used as a blank control.

### 2.3. Determination of the Solubility

To determine the solubility of the BBH using a standard curve or single spectrum, a measured amount of the BBH was added to an aqueous solution and mixed vigorously at 30 °C until the BBH was completely dissolved. The resulting suspension was then filtered through a Millipore filter with a pore size of 0.22 μm to remove any undissolved BBH. Next, a series of BBH standard solutions with known concentrations were prepared (Table A1 in Appendix A). These concentrations covered a range that allowed for unknown concentrations in the samples to be accurately determined. The prepared standard solutions were analyzed using a microplate reader (Thermo Fisher Scientific Multiscan-GO spectrophotometer, Hillsboro, OR, USA) at 263 nm, and a standard curve was established as described in Figure A2 in Appendix A.

### 2.4. Synthesis of CS/CMCD and Synthesis of BBH-Loaded CS/CMCD

**Figure 1:**
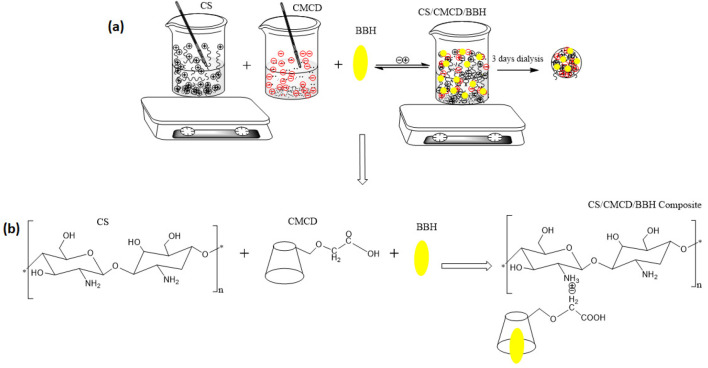
The CS/CMCD/BBH hydrogel composite formulation. (**a**) The physical interaction of CS/CMCD loaded BBH; (**b**) Chemical structure of CS, CMCD and CS/CMCD loaded BBH.

The CS/CMCD hydrogel was prepared using a physical cross-linking reaction through stirring until the hydrogel formed. Typically, the CS was dissolved in a 1% acetic acid solution, and the CMCD, at different ratios (10%, 8%, 6%, 4%, 2%, and 1%), was dissolved in deionized water. After thorough mixing, the CMCD solution was added to the CS solution, which was stirred at room temperature and allowed to react for a few minutes, as shown in Figure A3 in the Appendix A. The mixture was then placed in a dialysis membrane bag (MW: 3500.36 MM, with an atomic cutoff of 50 kDa) and dialyzed against distilled water for 5 days to remove unreacted reagents. The dialysate was replaced with deionized water every 4 h. Finally, the reactants were removed from the solution and freeze-dried for 48 h to obtain a white flocculated CS/CMCD composite hydrogel. To prepare the CS/CMCD hydrogel composite loaded with solid BBH, the lyophilization method was used, based on the molar ratio determined from the CS/CMCD synthesis studies. Briefly, 1% BBH was dissolved in water at 30 °C for 4 h and quickly filtered through a 0.22 µm pore size Millipore filter to remove any free BBH. This solution was then added to the CS/CMCD solution and the resulting BBH-loaded composite hydrogel was stirred continuously for 1 h before being slowly cooled to room temperature. The prepared CS/CMCD/BBH complex was then transferred to a dialysis bag and dialyzed against deionized water for 72 h, with the water replaced every 4 h to remove any unbound berberine hydrochloride. Then, the reagent was freeze-dried and the CS/CMCD/BBH hydrogel was obtained (Figure 1).

### 2.5. Characterization of the CS/CMCD and CS/CMCD/BBH Composite Biomaterials

Fourier transform infrared spectroscopy (FTIR) of the CS/CMCD and CS/CMCD/BBH composites was performed with a Fourier transform infrared spectrometer (Bruker VERTEX 70, Ettlingen, Germany). The samples (CS, CMCD, CS/CMCD/BBH, and CS/CMCD/BBH) were prepared using the dry KBr disk method. The experimental data were recorded in the range from 400 to 4000 cm^−1^. Furthermore, proton nuclear magnetic resonance analysis of the composite biomaterials was performed by a nuclear magnetic resonance spectrometer (AV300 NMR, Bruker, Fällanden, Switzerland) using a 1% solution of deuterium acetic acid (D_2_O) for dissolution, while the X-ray diffraction of the composite biomaterial dry powders was recorded by using an X-ray diffractometer (40 kV, 100 mA). In addition, thermogravimetric analysis of the samples was performed using a TGA spectrometer (TGA800, Perkin, Cambridge, MA, USA). The tensile properties of the CS/CMCD were tested using a mechanical performance testing machine (i-Strentek 1510 10 N (2.20 lbf)), with a diameter of 30 mm and a height of 1.28 mm, and the tensile properties were measured at 25 °C at 5 mm compression at a rate of 5 mm/minute, and the software automatically recorded the curve of force versus deformation. Finally, the morphology of the samples was observed using a FEI Sirion 200 scanning electron microscope. The determination of the water content (WDC) of the CS/CMCD was performed using the loss-on-drying method. The initial weight of the composite compound was measured, and the sample was then completely freeze-dried. The WDC was calculated as follows:

(1) $$\mathrm{WCD}\left(\%\right)=\left(\frac{iw}{dw}\right)\times 100$$

The (*iw*) is equivalent to the first weighing, and the (*dw*) is equivalent to the second weighing.

### 2.6. Drug Loading and Drug Delivery System

#### 2.6.1. Determination of the Loading Capacity and Encapsulation Efficiency

The loading capacity (LC) of the BBH in the chitosan/β-cyclodextrin hydrogel was determined by quantifying the amount of BBH entrapped in the hydrogel matrix. The encapsulation efficiency (EE) represents the percentage of BBH effectively encapsulated within the hydrogel matrix. Briefly, the CS/CMCD was weighed and placed in BBH at 45 °C for 24 h. Then, it was removed and placed under vacuum for 5 min to eliminate any excess BBH, weighed, and then dried overnight. After freeze-drying, the sample was weighed again. The loading capacity was calculated by dividing the amount of drug loaded into the hydrogel by the weight of the hydrogel itself. The encapsulation efficiency was calculated by dividing the amount of drug loaded into the hydrogel by the total amount of drug initially added and then multiplying by 100 to express it as a percentage [20]. The EE and LC of BBH were calculated according to the following formulae:

(2) $$\mathrm{EE}\%=\frac{\mathrm{wl}-w0}{\mathrm{wf}}\times 100\;$$

(3) $$\mathrm{LC}\%\equiv \frac{\mathrm{wl}-w0}{\mathrm{wl}}\times 100\;$$

where w0, wf, and wl are the amount of blank composite material, the weight of the initial drug, and the amount of the remaining drug, respectively.

#### 2.6.2. In Vitro Drug Loading and Drug Release

First, the BBH was prepared according to the method of Huang et al. [25] with slight modifications. Briefly, 1 mg/mL of BBH was dissolved in water, mixed, and slightly heated. The CS/CMCD composite was then added to the BBH solution and heated for 1 min at 45 °C. Then, the samples were frozen and dried for 40 h to obtain a loaded or charged CS/CMCD/BBH complex, as shown in Figure A5 in Appendix A. The dosage of BBH was calculated with a calibration curve of 0.1 μg/mL^−1^–10 mg/mL^−1^. The corresponding regression equation was (μg/mL^−1^) = −0.007 + 0.2929x, R^2^ = 0.9994 (where x is the absorbance of the drug, y is the drug concentration, and R^2^ is the drug loading concentration), as described in Figure 3. Second, the drug release curve of the BBH from the CS/CMCD composite was obtained using the rotating basket method at a speed of 100 revolutions per minute in the automatic dissolution apparatus, and the in vitro release curve at 37 °C was drawn. The oral drug delivery system is simulated. An acidic solution with a pH of 2.1 in the culture medium stimulated the gastric environment, a basic solution with a pH of 6.8 simulated the intestinal environment, and a neutral solution with a pH of 7.4 simulated the colon environment. For the rigor of the experiment, we also discussed the release behavior in alkaline media. The dissolution medium of 10 mg of drug was 9 mL of SGA/HCL solution, SIE/HCL solution, and SCE/phosphate buffer or NaOH solution (i.e., a pH of 2.1, 6.8, 7.4, and 12), and the temperature was maintained at 37 °C ± 0.5 °C. The sample (1 mL) was removed after 0.5 h, 1 h, 2 h, 4 h, 6 h, 8 h, 12 h, 24 h, 36 h, and 48 h, and immediately changed to 10 mL of the new preheating medium after 72 h. The maximum absorption wavelength of the drug was obtained by scanning the whole waveband.

### 2.7. Antimicrobial Assessment

Antibacterial tests were carried out on the CS, BBH, CS/CMCD/BBH, and CS/CMCD composites by the uniform diffusion method. In this study, the following three bacteria and yeasts were selected for the antibacterial testing: the Gram-negative bacteria *Escherichia coli* and the Gram-positive bacteria *Staphylococcus aureus* and *Candida albicans*. Microbial culture media were prepared with MHB and YPDA media at 37 °C. The inoculum density (10^5^ CFU/mL) of the broth culture solution was evaluated by an MC-Farland 0.5 standard solution, spectrophotometer, and 1:10 s dilution method. The surface of the agar plate was inoculated to distribute the microorganisms. The hydrogel was cut into small disks with a diameter of 1 cm and placed on agar plates. The agar plates containing the samples were labeled separately, placed upside down, and incubated at 37 °C for 24 h. After 24 h, the samples were removed and observed. The diameter of the zones of inhibition was recorded at 24, 48, and 72 h.

### 2.8. Cell Viability Cytotoxicity Assay

The cytotoxicity of the CS/CMCD composite was evaluated by the CCK-8 method (CK04 cell counting detection kit) in fibroblast and epithelial cell lines. The cells were seeded at a concentration of 1 × 10^4^ in a 96-well plate and incubated overnight. Then, different concentrations of the CS/CMCD hydrogel were added to each well. After incubating for 48 h, 10 μL of the CCK-8 solution was added to each well. After incubating for 30 min in a humid environment containing 5% CO_2_ at 37 °C, the absorbance was measured at 450 nm using a microplate reader (Multiscan G-O Thermo Fisher, Hillsboro, OR, USA). Cell viability was calculated using the following formula:

(4) $$\mathrm{Cell}\;\mathrm{viability}=\frac{\mathrm{OD}1-\mathrm{OD}2}{\mathrm{OD}3-\mathrm{OD}2}\times 100$$

### 2.9. Drug Efficacy Assay

The cytotoxicity of the BBH-loaded CS/CMCD hydrogel was evaluated in 3T3 cells and HaCaT cells. The cells were seeded in 96-well plates at 1 × 10^4^ cells per well and incubated at 37 °C overnight. The cells were then added to different formulations of the sample composite biomaterials and incubated for several minutes, after which 10 μL of CCK-8 solution was added to each well after incubation for 24, 48, or 72 h. After 30 min of incubation at 37 °C in a humidified atmosphere containing 5% CO_2_, the absorbance was measured at 450 nm using a microplate reader (Multiscan G-O Thermo Fisher, Hillsboro, OR, USA). The cell viability was calculated using the following formula:

(5) $$\mathrm{Cell}\;\mathrm{viability}=\frac{\mathrm{OD}1-\mathrm{OD}2}{\mathrm{OD}3-\mathrm{OD}2}\times 100$$

### 2.10. Confocal or Staining Assay

To confirm the cytotoxicity of the drug, the cellular uptake of the CS/CMCD/BBH composite into the NIH 3T3 cell line was studied. The NIH 3T3 cells (5 × 10^4^/well) were seeded on and incubated overnight. Then, the CS/CMCD/BBH composite (containing 5 μg/mL of BBH) was diluted in culture medium and incubated with the cells for 1 h at 37 °C. After the medium containing the CS/CMCD/BBH composite was removed, the cells were washed with PBS three times, stained with calcein AM solution for 15 min, and eventually washed three times with PBS. The sample was imaged with a confocal laser scanning microscope (Olympus (Tokyo, Japan), FV1000). The cellular uptake of the CS/CMCD hydrogel composite and free BBH was investigated as controls.

### 2.11. Statistical Analysis

All of the statistical analyses and measurements were performed using Origin PROLAB v. 2024b. The data obtained were analyzed using analysis of variance (ANOVA) in triplicate, and the results are expressed as the mean ± standard deviation.

## 3. Results

### 3.1. Wavelength Study and Solubility

**Figure A1:**
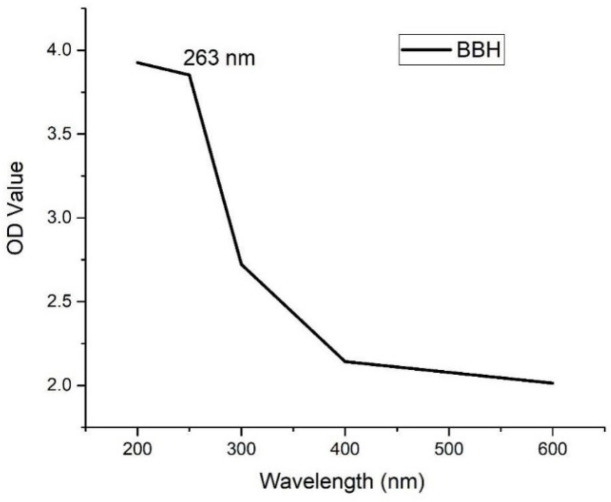
Selection of the detection wavelength of the BBH.

The wavelength study confirmed the characteristic absorption peaks of the BBH in the ultraviolet–visible (UV–Vis) spectrum. BBH is known to exhibit strong absorption at approximately 250, 350, and 430 nm in the UV–visible region of the electromagnetic spectrum [40]. The absorption spectrum of BBH typically shows two distinct peaks. The first peak occurs in the UV range (at approximately 260–280 nm), while the second peak occurs in the visible range (at approximately 350–400 nm). In the wavelength range of 200–400 nm, the absorption at 263 nm is stronger than that at 345 nm, so 263 nm was used as the detection wavelength. The scan result is shown in Figure A1 in Appendix A. This result is consistent with the work of Li et al. [8]. 

**Figure A2:**
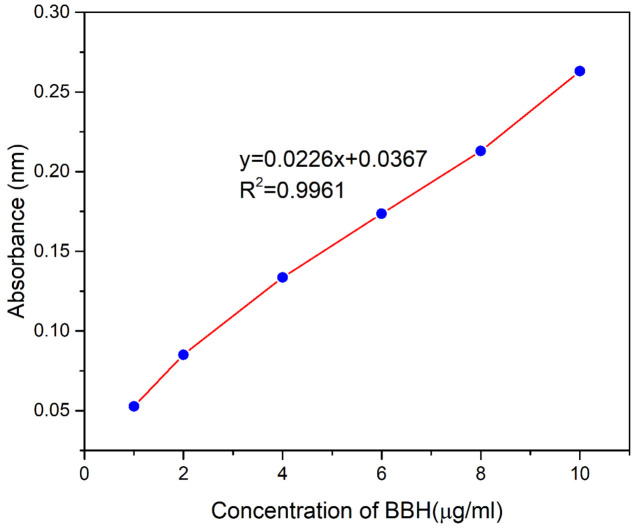
Phase solubility curve of the BBH in aqueous solution.

The solubilization effect of the BBH was evaluated by the solubility phase method [40]. The phase solubility diagram is shown in Figure A2 in Appendix A. The graph was created with the concentration (μg/mL) on the x-axis and the absorbance on the y-axis. The absorbance values obtained from the standard solutions at their corresponding concentrations are shown in Table A1 in Appendix A. Regression analysis was performed on the plotted data to determine the best-fit line that represents the relationship between the concentration and absorbance. The most common regression method for this purpose is linear regression. The equation of the line was calculated as y = mx + c, where y is the absorbance, x is the concentration, m is the slope, and c is the y-intercept. Once the standard curve was established, we used it to determine the concentration of an unknown sample of BBH by measuring its absorbance and applying the equation of the best-fit line obtained from the standard curve.

### 3.2. Synthesis of CS/CMCD and Synthesis of CS/CMCD-Loaded BBH Hydrogel

**Figure A3:**
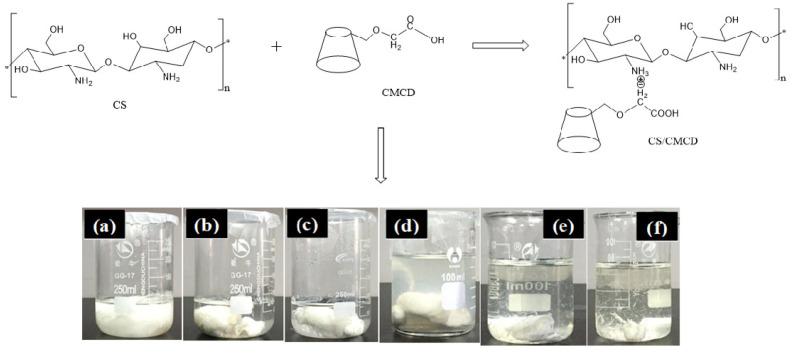
CS/CMCD composite hydrogel with different formations of CMCD: (**a**) 1% CS_1%CMCD, (**b**) 1% CS_2%CMCD, (**c**) 1% CS_4%CMCD, (**d**) 1% CS_6%CMCD, (**e**) 1% CS_8%CMCD, and (**f**) 1% CS_10%CMCD, respectively.

The synthesis of the CS/CMCD complex hydrogel involved the combination of CS and CMCD through physical cross-linking, followed by a freeze-drying process to form a stable network. The resulting hydrogel exhibited a three-dimensional network structure, with CMCD molecules trapped within the chitosan matrix (Figure A3 in Appendix A and Video S1). This structure gives the hydrogel properties such as a controlled drug release and high water absorption capacity (as shown in Table 1). The formation of hydrogels from highly concentrated and less concentrated solutions indicates strong physical cross-linking interactions between the CS and CMCD. The carboxymethyl groups of CMCD interact with the amino groups of chitosan via hydrogen bonds and electrostatic interactions, leading to the formation of physical cross-links between the polymer chains. The concentrations of CS and CMCD in the solution impact the gelation behavior and properties of the resulting hydrogel. Higher concentrations of both components generally result in the formation of stronger and stiffer hydrogels. The development and properties of the CS/CMCD hydrogel highlight its potential as an effective oral delivery system for BBH. The synthesis of the CS/CMCD hydrogel involved the cross-linking of CS and CMCD. BBH was subsequently loaded into the hydrogel matrix during the synthesis process. The synthesis was confirmed by various characterization techniques, which provided detailed insights into the structure and composition of the hydrogel and its BBH-loaded counterpart.

### 3.3. Characterization of CS/CMCD and CS/CMCD/BBH Hydrogels

#### 3.3.1. Analysis of Water Content

Water content determination (WCD) analysis is a process used to measure the amount of water present in a substance or sample [41]. The method used here was the loss-on-drying (LOD) method; this method involves weighing a sample before and after drying until a constant weight is reached. The weight loss corresponds to the water content of the sample. The weight of the product was recorded for each set of samples. The analysis of the water content of the CS/CMCD hydrogels showed a slight increase in water retention in the samples with a high concentration of CMCD, as shown in Table 1. This increase is attributed to the hydrophilic nature of CMCD, which improves the water retention capacity of the hydrogel matrix. Higher concentrations of CMCD result in a better WCD performance. Therefore, the higher water content is beneficial for the sustained release of BBH. 

#### 3.3.2. Proton NMR

**Figure 2:**
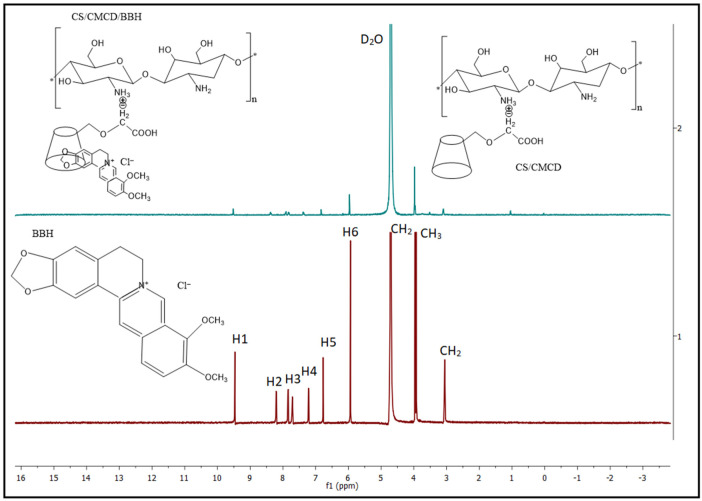
^1^HNMR spectra of the BBH and CS/CMCD/BBH (1%CS_6%CMCD_1%BBH) hydrogel.

The ^1^HNMR spectra of the CS, CMCD, and CS/CMCD hydrogels in 1% D_2_O are shown in Figure A2 in the Appendix A, while the ^1^HNMR spectra of the BBH and CS/CMCD/BBH composite hydrogel in 1% D_2_O are shown in Figure 2. The ^1^HNMR spectrum of CS shows one signal peak at 2.87 ppm, appearing at CH_3_ (N-glucosamine), and multiple signal peaks at 3.38 ppm, appearing at H-2 (glucosamine). H-3 to H-6, attached to non-anomeric carbon atoms, are adequate; the signal peak between 3.5 and 4.0 ppm is the signal peak of C3–C6, connected to the glucopyranose ring, and H-1 of the anomeric proton is at 4.8 ppm [33,42]. The ^1^HNMR spectrum of the CMCD shows multiple signal peaks for H-2, H-3, H-4, H-5 andH-6, with an apparent peak arrangement at 3.54 ppm, 3.96 ppm, 3.49 ppm, 3.76 ppm and 4.01 ppm respectively, and only one peak at 5.17 ppm due to the heteromeric protons of H-1 of α-D-pyranose [36,43]. The ^1^HNMR spectra of the CS/CMCD composite show that the multiple signal peaks at 3.38–4.34 ppm for the CS/CMCD composite were attributed to the H proton of CMCD and CS. The new signal at 1.82 was caused by the H proton of the subtitled CD H proton. Additionally, the peak reached 1.90 ppm for the glucosamine proton peak in the CS [20]. These observations confirm that the predicted CS/CMCD product was synthesized efficiently. The ^1^HNMR of BBH shows characteristic signal peaks at 9.47–7.21 ppm, which are attributed to the protons H-1 to H-6, while the peaks at 5.94 and 3.06 are attributed to CH_2_ and the peak at 4.02 is attributed to CH_3_. The ^1^HNMR spectrum of the CS/CMCD/BBH composite shows the peaks of multiple signals, with the peak at 3.97-3.10 ppm corresponding to the chemical shifts of the H protons of the CS and the CMCD. The peaks at 9.59–7.03 ppm are attributed to the H protons of the BBH, while the peak at 5.97 ppm is attributed to the CH_2_ proton of the BBH [44,45]. Further, the CMCD and BBH can form complexes with the CS, changing the chemical environment of the protons in a way that shifts them to higher fields or that reduces the total number of peaks. So, the presence of these characteristic peaks of BBH in the CS/CMCD/BBH spectra confirms the successful incorporation of the BBH into the hydrogel matrix and supports the enhanced solubility and stability of BBH in the oral formulation.

#### 3.3.3. FTIR

**Figure 3:**
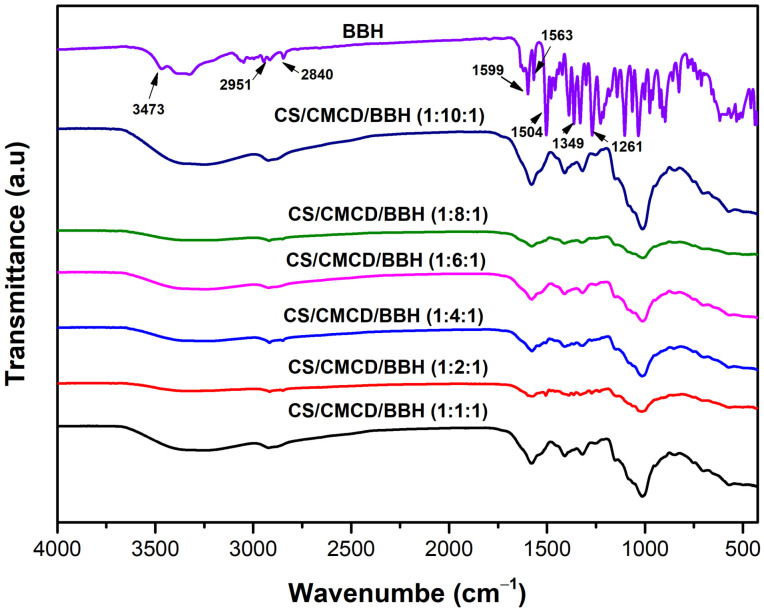
FTIR images of the BBH and CS/CMCD/BBH hydrogel with different formulations of CMCD.

**Figure A5:**
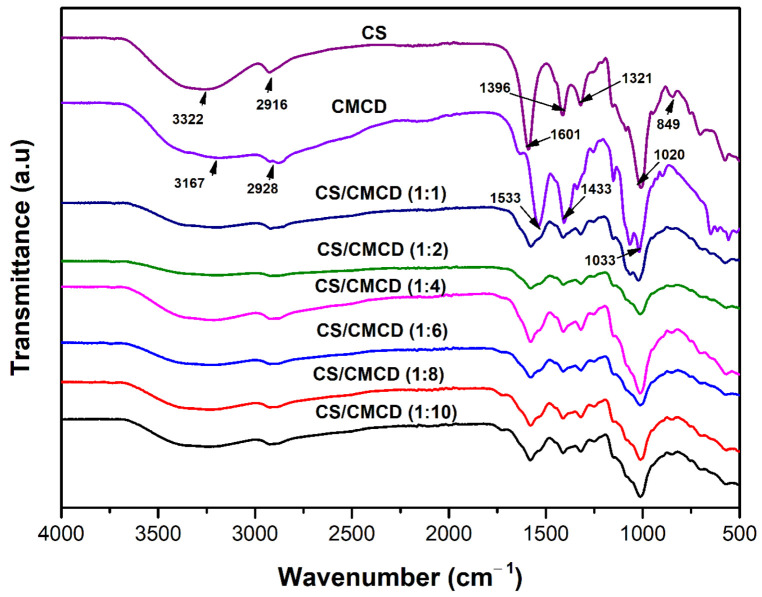
FTIR image of CS, CMCD, and CS/CMCD hydrogel with different formulations of CS/CMCD.

The FTIR spectra of the CS, CMCD, and CS/CMCD hydrogels with different CMCD formulations are shown in Figure A5 in the Appendix A. In the infrared spectrum of the CS, the peak at 3322 cm^−1^ corresponds to an amine symmetry vibration, while the peak at 2921 cm^−1^ is indicative of a stretching vibration of the CH. The peaks at 849 cm^−1^, 902 cm^−1^, and 1396 cm^−1^ are associated with the sugar structure of chitosan. Furthermore, the broad peak at 1020 cm^−1^ corresponds to the stretching vibration of CO. Other notable peaks include those at 1601 cm^−1^ and 1321 cm^−1^. Which are assigned to the amino proton group and the intermediate group, respectively. In the infrared spectrum of CMCD, broad absorption peaks at 2928 cm^−1^ and 1433 cm^−1^ are assigned to the -OH and -C-O groups. The broad absorption peak at 3000 cm^−1^ is due to the hydroxyl group. The spectrum of the CS/CMCD hydrogel shows the α-pyranose vibration of CMCD at 1033 cm^−1^ and the secondary amide band of chitosan at 1601 cm^−1^ [46]. These results confirm the successful synthesis of the CS/CMCD hydrogel. The FTIR spectra of the BBH and CS/CMCD/BBH hydrogel are displayed in Figure 3. The BBH spectrum shows characteristic peaks at 3473, 2951, 2840, 1616, 1599, 1579, 1504, 1349, 1261, 1233 cm^−1^. The peak at 1616 cm^−1^ is identified as the quaternary ammonium ion C=N [47,48]. Comparing the spectra of the BBH with the CS/CMCD/BBH hydrogel, a notable shift in the protons from the C=N peak from 1616 cm^−1^ to 1670 cm^−1^ is observed. In addition, the C–O vibration peak disappeared and new peaks appeared in the range of 500–600 cm^−1^ due to the C skeletal vibrations. Moreover, the peak intensity between 2000 and 3000 cm^−1^ decreased, indicating a change in the IR range within the complex. These spectral changes suggest strong interactions between the BBH and the hydrogel matrix [49]. Therefore, the FTIR analysis provides strong evidence for the successful synthesis of the CS/CMCD hydrogel and the efficient encapsulation of the BBH in this matrix. The observed spectral shifts and changes indicate significant interactions between the BBH and hydrogel components, which are crucial for improving the solubility and stability of BBH for oral administration. 

#### 3.3.4. XRD

**Figure 4:**
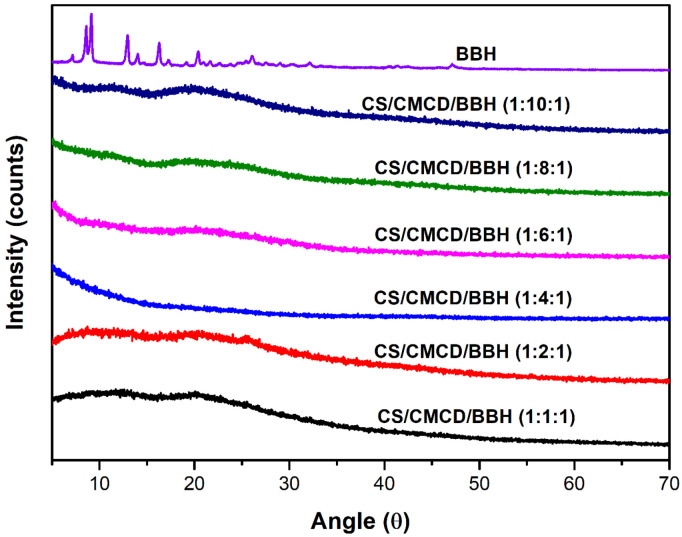
XRD images of the BBH and CS/CMCD/BBH hydrogel with different formulations of CMCD.

**Figure A6:**
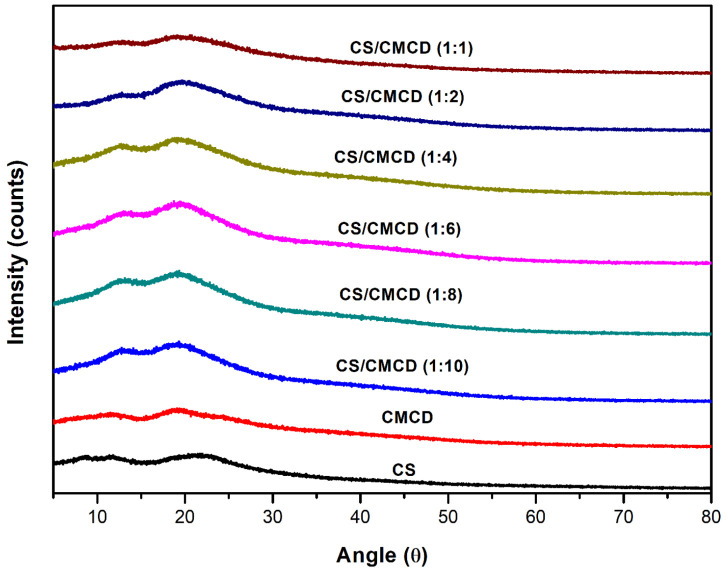
XRD image of CS, CMCD, and CS/CMCD hydrogel with different formulations of CMCD.

The XRD analytical was used to assess the crystallinity of the samples. The XRD patterns of the CS, CMCD, and CS/CMCD hydrogels with various CMCD formulations are shown in Figure A6 in the Appendix A. The XRD patterns of the BBH and CS/CMCD/BBH hydrogel are shown in Figure 4. The XRD profile of the CS displays distinct crystalline peaks at approximately 2θ = 10–11° and 19–22°, corresponding to the presence of amine I (-N-CO-CH_3_) and amine II (-NH_2_) groups, respectively. These peaks confirm the crystalline nature of the CS, which is essential for its structural integrity [50]. While, the XRD pattern of the CMCD exhibits characteristic intense peaks in the range from 15° to 30°, reflecting its crystal structure [22]. When the CMCD was incorporated into the CS matrix to form the CS/CMCD hydrogel, the XRD pattern demonstrated a shift toward a more amorphous structure, as indicated by the broadened peaks around 2θ = 7.90–10° and 2θ = 21.0°. This change in the diffraction pattern suggests that the interaction between the CS and CMCD reduced the crystalline order of the materials, resulting in a hydrogel with increased amorphous characteristics. The transition from a crystalline to an amorphous structure in the CS/CMCD hydrogel is significant, as it indicates the successful incorporation of the CMCD into the CS backbone, leading to the formation of a new material with distinct physical properties. The increased amorphous nature of the hydrogel enhances its ability to encapsulate and disperse active pharmaceutical ingredients, such as BBH, within its matrix. The XRD pattern of the BBH alone showed distinct crystalline peaks at 2θ = 8.6°, 9.1°, 12.9°, 16.2°, 20.9°, 25.4°, 30.5°, and 45.3°, illustrating its crystalline nature. However, when the BBH was incorporated into the CS/CMCD hydrogel, these characteristic peaks were either diminished or broadened, indicating a transition to a more amorphous structure. This amorphization is crucial because it suggests that the BBH is well dispersed within the hydrogel matrix, reducing its crystallinity. The enhanced amorphous nature of the CS/CMCD/BBH hydrogel is significant because it contributes to the increased solubility of BBH, an essential factor for its effectiveness in oral administration [51,52].

#### 3.3.5. TGA

**Figure 5:**
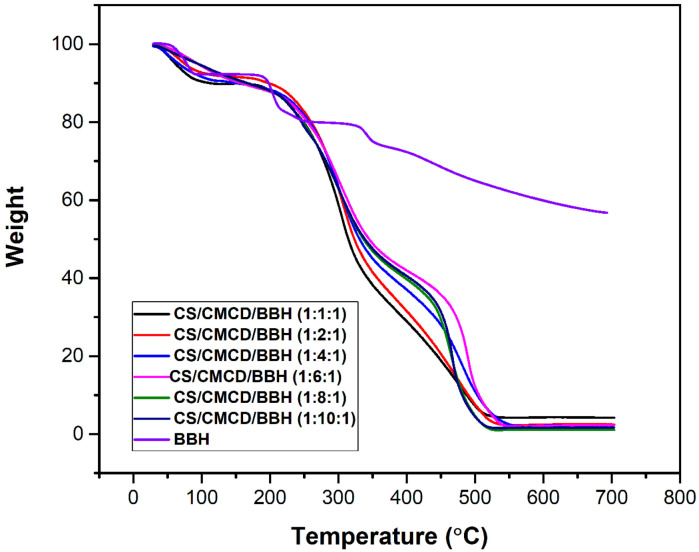
TGA of the BBH and CS/CMCD/BBH hydrogel with different formulations of CMCD.

**Figure A7:**
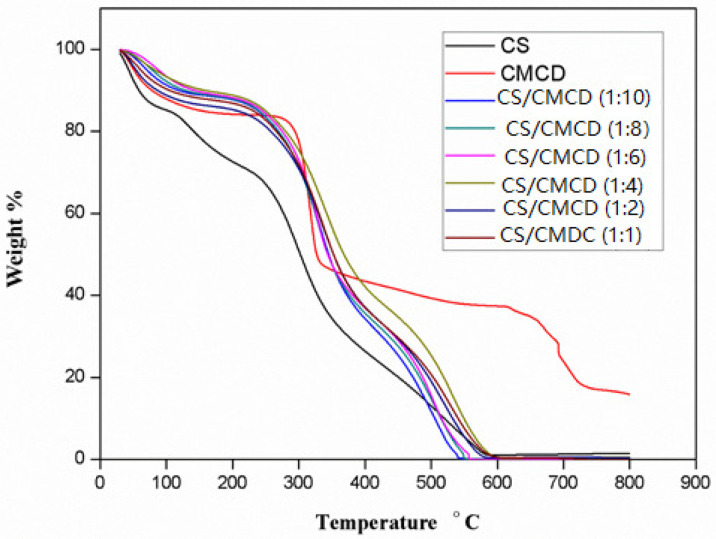
TGA image of CS, CMCD, and CS/CMCD with different formulations of CS/CMCD.

The thermal stability of t CS, CMCD, and CS/CMCD hydrogels are shown in Figure A7 in the Appendix A. The TGA of the BHH and the CS/CMCD/BBH hydrogel are shown in Figure 5. The TGA of the CS typically exhibits weight loss in two stages. The TGA curve of the CS shows weight loss in the first stage, occurring between shows a weight loss in the first stage 46–125 °C, with a weight loss of 10.2% due to the loss of residual or physically adsorbed water on the membranes’ surfaces. Then, the weight loss starts at 295 °C and continues up to 545 °C, during which there was 41.5% weight loss due to the degradation of the CS. The TGA curve of the CMCD shows a weight loss in three stages. The first stage starts under 100 °C, with a 14.8% mass loss that can be attributed to the evaporation of superficial water associated with the cyclodextrin. The second stage, at around 110 °C, with a 10% mass loss, can be attributed to the evaporation of internal water [53]. Then, the third stage, at around 307 °C, can be related to the degradation of the β-CD [54]. At this point, 65.7% of the mass is reduced. The TGA curve of the CS/CMCD hydrogel shows a weight loss in two stages. The first stage starts with a weight loss in the range from 25 to 258 °C, with 8% of the adsorbed and bound water weight loss on the membranes’ surfaces. The second stage of the weight loss starts from 275 to 742 °C, during which there is a 37.5% weight loss due to the degradation of chitosan. There is 29.5% weight loss observed in the ranges from 750 to 995 °C, which contributes to the decomposition of the CMCD in the CS/CMCD interaction, as indicated by the high degree of thermal stability of the composite. The CS/CMCD hydrogel might improve the thermal stability through hydrogen bonding and electrostatic attraction interactions. The TGA curve for the BBH exhibits weight loss in four stages. The initial weight loss of the BBH starts at 109 °C, with an 8.3% weight loss attributed to the evaporation of moisture, or which corresponds to the loss of moisture [55]. The second stage starts between 109 and 188 °C, with a 1.3% weight loss, signifying the melting temperature of the BBH. The third stage showed a 20.6% weight loss at 250 °C, revealing the decomposition of the BBH [39]. The fourth stage, in the 250–790 °C range, was ascribed to the destruction of the BBH skeleton structure. In the TGA curve for the CS/CMCD/BBH hydrogel, the maximum weight loss occurred at about 265 °C, with an 18.3% weight loss reached at approximately 500 °C, with the disintegration finished at 549 °C, showing that the CS/CMCD/BBH hydrogel displays great intensity obstruction. However, the TGA curves demonstrated that the CS/CMCD/BBH hydrogel had a slightly lower decomposition temperature compared to the CS/CMCD hydrogel, likely due to the presence of BBH. Additionally, the curves showed that the thermal stability of the BBH-based polymer was not impacted by the temperature during the process. The TGA curves demonstrated that the CS/CMCD/BBH hydrogel had a slightly lower decomposition temperature compared to the CS/CMCD hydrogel, likely due to the presence of BBH. However, the thermal stability remained adequate for potential oral administration applications.

#### 3.3.6. Mechanical Properties

**Figure A8:**
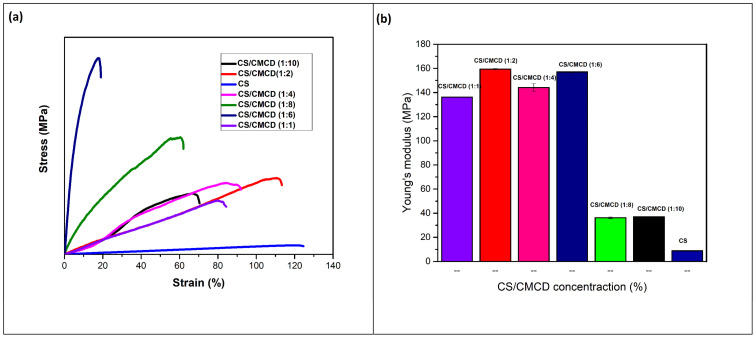
Mechanical properties of CS and CS/CMCD hydrogel with different formulations of CMCD. (**a**) Tensile assay; (**b**) Young’s modulus.

Tensile tests were conducted to evaluate the mechanical properties of the CS/CMCD hydrogels, as shown Figure A8 in the Appendix A, with an emphasis on the compressive strength, toughness, and Young’s modulus. The hydrogels exhibited exceptional mechanical properties, including high compressive strength and toughness, with no fractures observed even at a 100% strain for all of the samples tested. The results suggest that the hydrogels maintain excellent structural integrity under significant mechanical stress, which is essential for their application as drug delivery systems. Young’s modulus data, which measure the stiffness of the hydrogels, showed variation as a function of the CS/CMCD ratio. Specifically, Young’s modulus increased significantly as the CMCD content increased, reflecting the increased mechanical strength due to the greater chemical cross-linking and hydrogen bonding. For example, Young’s modulus reached 159.3 MPa with a CS/CMCD ratio of 1:2, and 157.1 MPa with a ratio of 1:16, compared to only 37.01 MPa for the pure CS. This substantial increase highlights the role of CMCD in strengthening the hydrogel matrix, making it more resilient to mechanical deformation. Interestingly, a lower Young’s modulus was observed at a CS/CMCD ratio of 1:8 and 1:10, with values of 36.2 and 37.01 MPa, which may indicate a critical point where the balance between the cross-linking and flexibility of the matrix is optimized, allowing for higher toughness but lower rigidity. The highest stiffness was observed with CS/CMCD ratios of 1:2 and 1:16, suggesting that these formulations are particularly suitable for applications requiring strong mechanical integrity. Despite the variability in the stiffness, the overall mechanical properties of the CS/CMCD hydrogel remain within acceptable ranges for oral formulations. This ensures that the hydrogels are robust enough to retain their structure while effectively delivering the drug, even under the mechanical stresses encountered in the gastrointestinal tract.

#### 3.3.7. SEM

**Figure 6:**
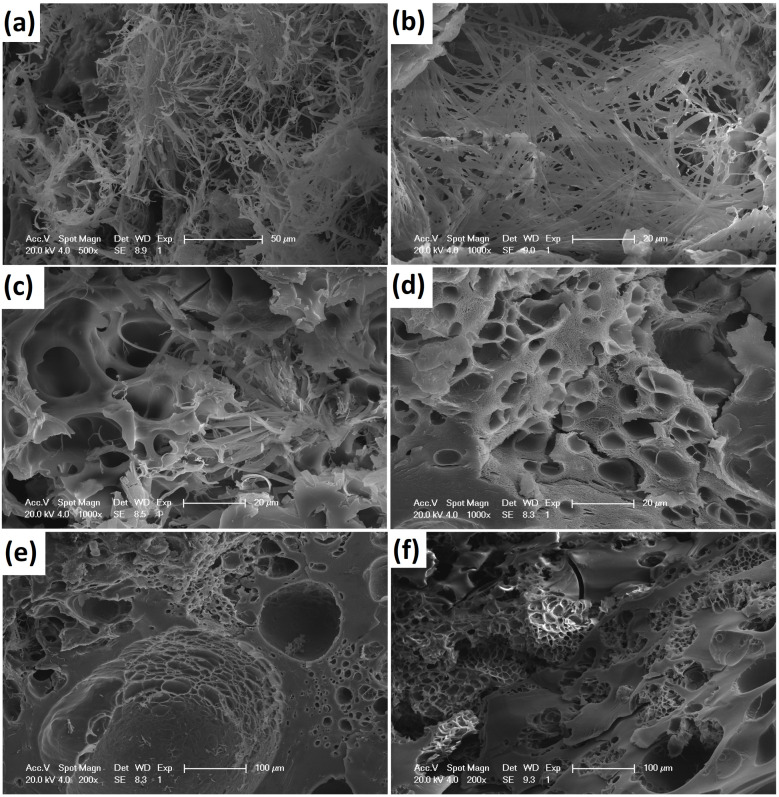
SEM image of the CS/CMCD/BBH hydrogel with different formulations of CMCD, with scale bars equal to 20 μm and 200 μm: (**a**) 1% CS_1%CMCD_1%BBH, (**b**) 1% CS_2%CMCD_1%BBH, (**c**) 1% CS_4%CMCD_1%BBH, (**d**) 1% CS_6%CMCD_1%BBH, (**e**) 1% CS_8%CMCD_1%BBH, and (**f**) 1% CS_10%CMCD_1%BBH.

**Figure A9:**
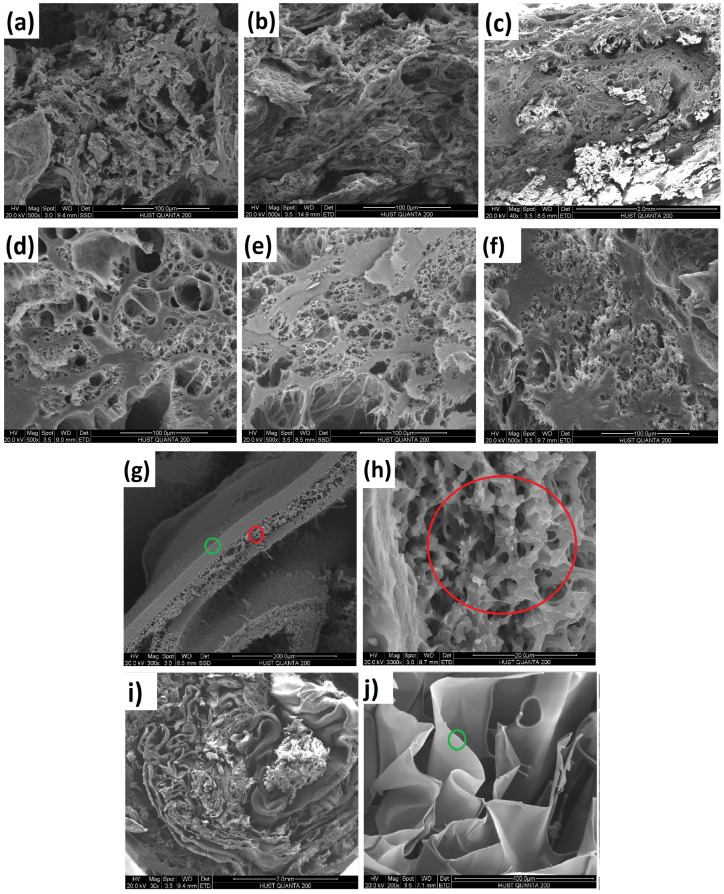
SEM image of CS/CMCD composite with different formulations of CMCD: (**a**) 1% CS_1%CMCD, (**b**) 1% CS_2%CMCD, (**c**) 1% CS_4%CMCD, (**d**) 1% CS_6%CMCD, (**e**) 1% CS_8%CMCD, and (**f**) 1% CS_10%CMCD; (**g**–**j**) image illustrating the cross-linking reaction of the CS/CMCD interaction circled in red and circled in green is CS, with the scale bar equal to 100 μm, 50 μm, and 400 μm.

The SEM images of the CS/CMCD hydrogel (Figure A9 in the Appendix A) and the CS/CMCD/BBH hydrogel (Figure 6) offer valuable insights into the microstructural characteristics of these materials. Compared with the CS/CMCD/BBH hydrogel, shown in Figure 6, the gel structure of the CS/CMCD/BBH hydrogel exhibited a looser gel structure, increased wall thickness, and more uneven pore sizes. The layered gel structure was more pronounced, forming a dense, uniform, and orderly hydrogel network. This indicates that the introduction of the BBH into the hydrogel matrix affected the overall structural organization, likely due to the interaction between the drug molecules and the hydrogel components. Despite these changes, the hydrogels maintained a dense, uniform, and orderly network, crucial for effective drug encapsulation and release. However, there was no significant difference between the microstructures. Notably, as seen in Figure A9 (Appendix A), the pore size increased with the concentration of CMCD, but all of the hydrogels maintained an interconnected network structure. This increase in pore size is significant because larger pores can facilitate the diffusion and sustained release of the drug, while the interconnected network ensures a consistent drug release profile. Figure A9b (Appendix A) illustrates the interaction between the CS and CMCD components, revealing a network of interconnected pores throughout the hydrogel matrix. This network structure is indicative of successful cross-linking and interaction between the CS and CMCD, which is vital for maintaining the integrity of the hydrogel under physiological conditions [56,57]. Furthermore, the hydrogels prepared in this study demonstrated improved performance, which is essential for efficient drug loading and release, compared to the hydrogels prepared in similar studies in which cross-linkers were added, and which still exhibited poor mechanical properties and signs of toxicity [58,59]. This improvement is crucial for their potential application in drug delivery systems, providing a more reliable and efficient method for the oral administration of BBH [60]. However, looking forward, although the current SEM analysis provides a qualitative understanding of the hydrogel microstructure, there is an opportunity to further improve our understanding through quantitative assessments. Future studies could benefit from using morphometry or porosimetry techniques to quantitatively assess the pore size, distribution, and overall porosity.

### 3.4. Drug Release and Loading System

#### 3.4.1. Loading Capacity and Encapsulation Efficiency

The loading capacity (LC) and encapsulation efficiency (EE) of the BBH in the CS/CMCD hydrogel were calculated and presented based on the experimental data obtained from the analytical methods described below. The results indicate the ability of the hydrogel to effectively trap and deliver BBH. The LC and EE values for different CS/CMCD ratios ranged from 84 to 103.07 and the EE values ranged from 100 to 121, as shown in Table 2. The loaded weight of the CS/CMCD was 10 times greater than the initial weight, and the highest LC of 103.07 and EE of 121 were observed for the CS/CMCD ratio of 1:10, indicating a higher drug loading capacity and retention in the hydrogel matrix at this ratio. These high values suggest that this formulation is particularly effective in encapsulating and retaining BBH, which could lead to better therapeutic outcomes through increased drug availability and controlled release. Higher CL and EE values directly correlate with a greater drug loading capacity and better drug retention, which are critical factors in the effectiveness of drug delivery systems. The improvement in these parameters suggests that the CS/CMCD hydrogel is very effective in improving the solubility, stability, and controlled release of BBH, thus enhancing its therapeutic potential. The results highlight the potential of the CS/CMCD hydrogel as a promising drug delivery system for BBH. The formulation’s ability to achieve high LC and EE values makes it a valuable tool for improving the bioavailability and therapeutic efficacy of BBH, paving the way for further development and optimization of this drug delivery system to hydrogel bases.

#### 3.4.2. Drug Release Profile

**Figure 7:**
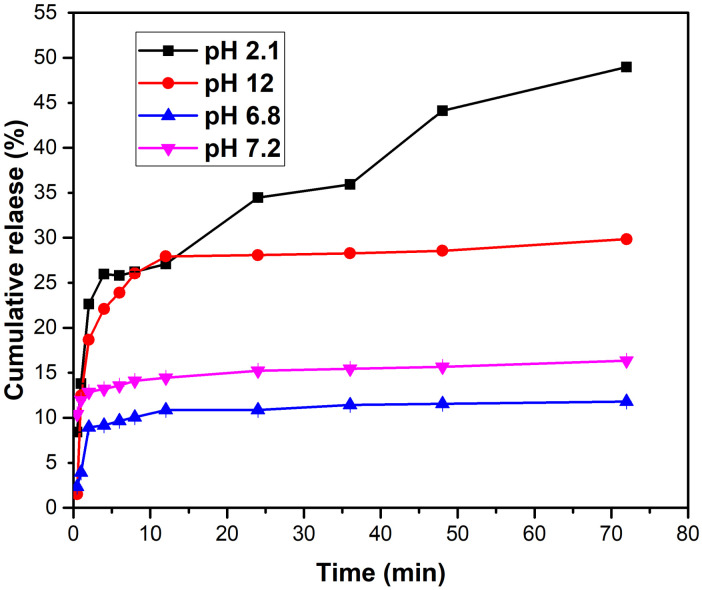
The release profile of the BBH solution at different pH values at room temperature.

Drug release studies are conducted to study how a drug is released from a particular delivery system over time [61,62,63]. In this study, the in vitro release of BBH from the CS/CMCD hydrogel was evaluated using the basket method by simulating body fluids and performing periodic sampling to measure the concentration of the drug released [64]. The environments used were simulated gastric fluid (SGF) with a pH of 2.1, simulated intestinal fluid (SIF) with a pH of 6.8, simulated colon fluid (SCF) with a pH of 7.2, and simulated basic liquid (SBF) with a pH of 12. As shown in Figure 7, the drug release patterns in all of the simulated fluids exhibited the following two distinct stages: an initial abrupt release followed by a sustained release or extended diffusion-controlled release. The initial abrupt release can be attributed to the presence of the drug on the hydrogel surface and the high concentration gradient of the drug, which acts as a driving force to release the drug from the hydrogel matrix. Following this burst release, the drug was released steadily over time, likely due to the formation of inclusion complexes between the drug and CMCD. The hydrogel in the SIF showed a lower release efficiency, with only 10% released over 24 h. In the SCF, approximately 15% of the BBH was released within 24 h, while, in the SBF, the release was approximately 35%. The SGF posted a 50% release in the same period. The release rate of the BBH was the fastest in the SGF, followed by the SBF, SIF, and finally SCF. The faster release of the SGF could be due to the protonation of the CS in the acidic medium, which increases the swelling of the hydrogel composite matrix, making its structure looser and allowing for a faster drug release. Conversely, the slower release in the SCF could be due to the formation of inclusion complexes between the CMCD and drug molecules, resulting in a slower drug release rate. Therefore, the sustained release of the BBH from the CS/CMCD hydrogel was effectively modulated, thereby improving its bioavailability and therapeutic efficacy. This controlled release profile highlights the potential of the CS/CMCD/BBH hydrogel as a viable drug delivery system for oral administration, addressing the solubility and delivery challenges associated with BBH. The release profile of the berberine hydrochloride (BBH) solution at different pH values matches the expectations based on similar studies [36].

### 3.5. Antibacterial Effect of CS/CMCD and CS/CMCD/BBH Hydrogels

**Figure 8:**
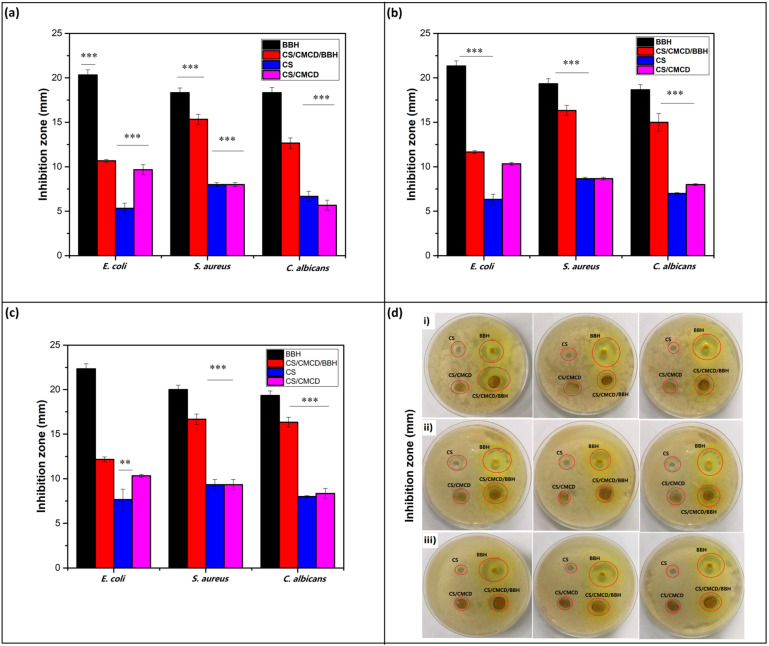
Antibacterial effect of the (**a**) twenty-four-hour, (**b**) forty-eight-hour, and (**c**) seventy-two-hour invasion zones of the samples: (**d**) *E*. *coli, S*. *aureus*, and *C*. *albicans* after 24 h of incubation. Mean ± SD (n = 3). (**) indicates *p*-value < 0.05, (***) indicates *p*-value < 0.005.

The antibacterial activities of the CS/CMCD and CS/CMCD/BBH hydrogels against *E. coli, S. aureus*, and *C. albicans* were evaluated using the agar diffusion method. As shown in Figure 8 and Table A2, Table A3 and Table A4 in the Appendix A, the CS/CMCD hydrogel demonstrated reduced antibacterial activity against *E. coli*, *S. aureus*, and *C. albicans* compared to the CS alone. However, the CS/CMCD/BBH hydrogel exhibited significantly better antibacterial activity against these pathogens. This increased activity is likely due to the antibacterial properties of the BBH, which disrupt the structure and function of microbial cells, interfere with their DNA, and inhibit various enzymes [2]. The inhibition zone areas for *E. coli*, *S. aureus*, and *C. albicans* were significantly larger for the CS/CMCD/BBH hydrogel compared to the CS/CMCD hydrogel. The sustained release of the BBH from the hydrogel into the solid medium contributed to this effect. As shown in Figure 8b, the CS/CMCD/BBH hydrogel maintained good antibacterial activity against *E. coli* for 24 h, with inhibition zone diameters greater than 10 mm, and the inhibition persisted for 48 and 72 h, as shown in Table A2, Table A3 and Table A4 in the Appendix A. Similarly, the hydrogels showed more pronounced inhibitory regions against *S. aureus* and *C. albicans*, with inhibition diameters greater than 15 mm on the first day and lasting antibacterial effects for up to three days. Even after three days, the hydrogel still demonstrated significant inhibition compared to the CS alone. The data suggest that the BBH is well-permeated into the hydrogel, resulting in a longer-lasting antibacterial effect. Therefore, the CS/CMCD/BBH hydrogel exhibited sustained antibacterial action for three days against *E. coli*, *S. aureus*, and *C. albicans* on agar plates. This sustained antibacterial activity is attributable to the inherent properties of the BBH, making the formulation suitable for the treatment of infections requiring prolonged antibacterial action.

### 3.6. Cytotoxicity Effect of CS/CMCD and CS/CMCD/BHH Hydrogels

**Figure 9:**
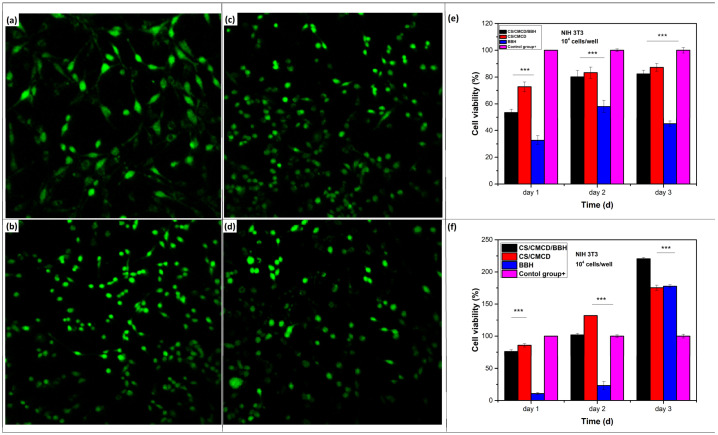
Cytotoxicity effect of the CS/CMCD/BBH hydrogel on NHI3T3 and HaCat cells. (**a**) Live and dead staining for the samples: (**a**) CS/CMCD, (**b**) CS/CMCD/BBH, (**c**) BBH, and (**d**) Control at twenty-four hours of incubation. (**e**) Cell viability through the solvent extraction of the samples. (**f**) Cell viability in direct contact with the samples. (***) indicates *p*-value < 0.005.

**Figure A10:**
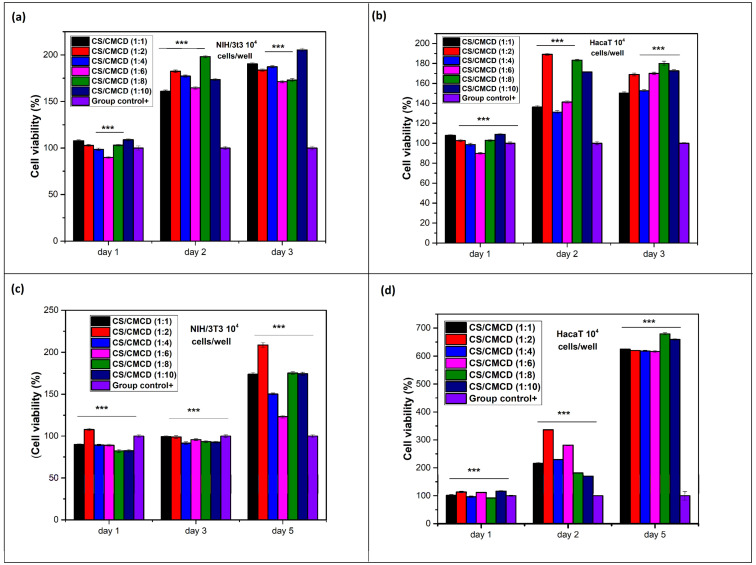
Cytotoxicity of CS/CMCD composite with different formulations of CMCD performed on NIH3T3 cells and HaCaT cells. (**a**,**b**) through the solvent extraction method, (**c**,**d**) in direct contact with the samples. (***) indicates *p*-value < 0.0005.

The cellular toxicity of CS/CMCD and CS/CMCD/BBH hydrogels was studied to evaluate their biocompatibility. Fibroblast cells (NIH/3T3) and keratinocyte cells (HaCaT) were cultured on scaffolds, and their cellular cytotoxicity was assessed using live/dead assays and the quantification of the cell proliferation via CCK-8 trials. Cells were directly cultured on the samples and on extraction media derived from the samples. The CCK-8 assay is commonly used to quantify the cell viability of cells growing on or inside of bioengineered scaffolds [65]. To check the initial cell viability of BBH, CS/CMCD and CS/CMCD/BBH, live/dead assays were performed (Figure 9). As shown in Figure 9a, live NIH3T3 cells exhibited green fluorescence when treated with calcein AM. The CS/CMCD/BBH hydrogel showed almost no dead cells. After 24 h of seeding on the CS/CMCD/BBH hydrogel, the cells were both alive and well-attached to the samples. Furthermore, the cells adhering to the CS/CMCD hydrogel exhibited a stretched morphology after 24 h, indicating the high cellular affinity of the CS/CMCD hydrogel. Cell proliferation on each sample was confirmed after 3 to 5 days of culture under direct contact and extraction conditions. As shown in Figure 9b and Figure A10 in Appendix A, the cell proliferation rate was compared to the 1-day control group (free cells) for quantification. The CS/CMCD/BBH hydrogel showed a significant difference in proliferation rates compared to the control group on day 1 of culture, with a 30% higher rate in solvent extraction culture than in direct contact culture. There was no significant difference in the proliferation rates between the CS/CMCD/BBH group and the control group in the direct contact culture, with a difference of only 22%. This trend continued for 2 days. However, on day 3, there was a significant difference in the proliferation rates between the CS/CMCD/BBH group and the control group, with the CS/CMCD/BBH group showing an increase of more than 100%. For the CS/CMCD hydrogel, as shown in Figure A10 in Appendix A, there was no significant difference in the proliferation rates compared to the control group on day 1, with only an 11% difference. This trend continued through 5 days, except for a significant difference on day 3, where the CS/CMCD group showed a 25% increase compared to the control group. No statistical differences were found between the two groups for the remainder of the testing period, indicating that, although the CS/CMCD hydrogel modification may slightly alter its physicochemical properties, it does not induce cytotoxic effects. These results demonstrate that the CS/CMCD/BBH hydrogel is biocompatible and safe for use at therapeutic concentrations, highlighting its potential for safe oral administration.

## 4. Discussion

In this study, an innovative hydrogel of chitosan (CS) and carboxymethyl-β-cyclodextrin (CMCD) was developed by the physical cross-linking to serve as an oral formulation for berberine hydrochloride (BBH). This formulation improves the solubility of BBH and addresses the limitations of its suitability for oral administration. The results demonstrated that the CS/CMCD composite was successfully prepared and synthesized with a high efficiency. An increase in the number of negative protons in ^1^HNRM improved the quality of the composite. The FTIR analysis indicated intermolecular interactions within the hydrogel, which contribute to its stability. The SEM images revealed clear evidence of a three-dimensional porous structure, crucial for facilitating drug loading and release. The XRD and TGA characterizations further confirmed that the synthesized CS/CMCD hydrogel significantly improved the solubility of the BBH. The mechanical properties of the hydrogel were consistent with these results, demonstrating sufficient robustness for oral administration. The loading capacity (LDC) studies showed that the BBH was efficiently loaded and incorporated into the CS/CMCD matrix, achieving a maximum encapsulation efficiency (EE) of up to 90%, indicating the high drug loading potential of the hydrogel. The swelling behavior of the hydrogel in water was also favorable, reaching a rate of up to 50%, which is important for the sustained release of BBH. The release profile of the BBH from the hydrogel varied across different pH levels, suggesting that the formulation can be tailored for targeted release in specific regions of the gastrointestinal tract. The hydrogel also showed a good swelling rate in water of up to 50%. The prepared hydrogel demonstrated the slow and sustained release of the BBH, with the drug release profile varying under different pH values. Additionally, the biocompatibility testing showed that the CS/CMCD/BBH hydrogel did not significantly impact the cell viability over five days, with evidence of cell proliferation after 24 h. The hydrogel also demonstrated notable antibacterial activity, particularly against *S. aureus* and *E. coli*, with sustained inhibition observed over days and with a maximum inhibitory diameter of 28 mm and 10 mm on *S. aureus* and *E. coli*, respectively. These results suggest that the prepared hydrogel can serve as a novel controlled drug release carrier and a promising material for drug delivery systems, including antibacterial wound dressings. 

## 5. Conclusions

The development of a CS/CMCD hydrogel as an oral delivery system for BBH represents a significant advance in overcoming the limitations of the low solubility and stability of BBH. This study demonstrates that this physically cross-linked hydrogel not only improves the solubility and bioavailability of BBH but also ensures sustained drug release, excellent biocompatibility, and effective antibacterial properties. The CS/CMCD hydrogel shows promise as a multifunctional material for various biomedical applications, including controlled drug release and antibacterial treatments. Additionally, although this study provides a solid basis for the use of CS/CMCD hydrogels in drug delivery, further research is needed to optimize the formulation parameters and explore other potential applications, such as the long-term stability and the evaluation of the safety profile of CS/CMCD hydrogels for BBH delivery. Additionally, conduct in vivo studies to evaluate the in vivo studies to evaluate the pharmacokinetics, cytotoxicity, bioavailability, and therapeutic efficacy of the hydrogel in a physiological environment. This will provide critical information for their future clinical applications.

## Figures and Tables

**Table 1:** Water content determination of the CS/CMCD hydrogels.

Sample (g)	First Weighing (g)	Second Weighing (g)	Water Content (g)	WCD (%)
CS/CMCD (1:10)	3.972 ± 0.005	1.211 ± 0.005	2.760 ± 0.001	69.5
CS/CMCD (1:8)	3.567 ± 0.0005	1.198 ± 0.005	2.369 ± 0.001	66.4
CS/CMCD (1:6)	3.248 ± 0.005	1.192 ± 0.005	2.056 ± 0.001	63.3
CS/CMCD (1:4)	2.989 ± 0.005	1.172 ± 0.005	1.817 ± 0	60.1
CS/CMCD (1:2)	0.937 ± 0.005	0.394 ± 0.005	0.539 ± 0.001	57.5
CS/CMCD (1:1)	0.195 ± 0.005	0.112 ± 0.005	0.083 ± 0	42.4

**Table 2:** Determination of LC and EE of different CS/CMCD ratios loaded with BBH.

Sample (mg)	Hydrogel Weight (mg)	Loaded Weight (mg)	Drug Weight (mg)	LC (%)	EE (%)
CS/CMCD (1:10)	22.44 ± 5.38	325.36 ± 0.44	85.62 ± 0.14	103.06 ± 4.41	121.79 ± 5.21
CS/CMCD (1:8)	18.6 ± 2.57	260.9 ± 4.17	242.3 ± 1.656	92.3 ± 0.86	100.7 ± 5.15
CS/CMCD (1:6)	17.16 ± 1.01	297.64 ± 0.76	253.32 ± 4.07	95.42 ± 3.37	112.77 ± 1.98
CS/CMCD (1:4)	16.80 ± 2.22	293.92 ± 4.00	248.7 ± 04.06	94.28 ± 3.30	111.42 ± 1.90
CS/CMCD (1:2)	16.28 ± 1.06	286.44 ± 7.34	231.78 ± 0.38	91.91 ± 1.19	108.62 ± 3.77
CS/CMCD (1:1)	15.55 ± 0.20	265.32 ± 0.23	221.34 ± 0.36	84.97 ± 1.05	100.42 ± 3.61

**Figure A4:**
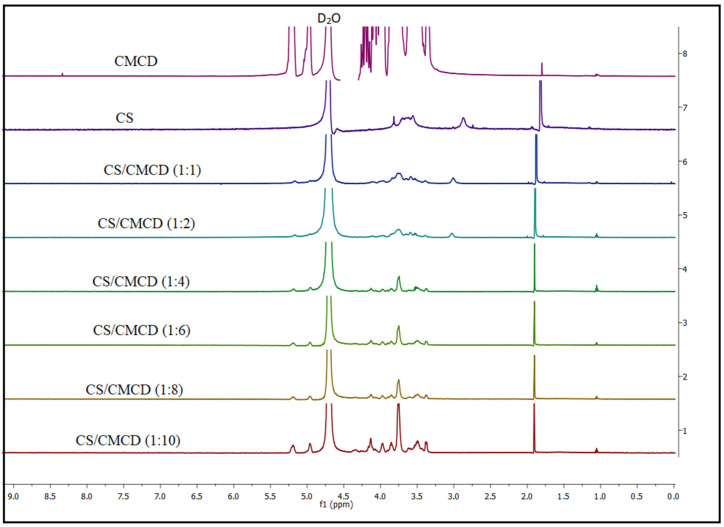
^1^HNMR spectra of CS, CMCD, and CS/CMCD hydrogel with different formulations of CS/CMCD.

## Data Availability

The original contributions presented in the study are included in the article and Supplementary Materials, further inquiries can be directed to the corresponding author.

## Supplementary Materials

The following supporting information can be downloaded at: https://www.mdpi.com/article/10.3390/polym16162368/s1, Video S1: CS/CMCD hydrogel.

## Appendix A

**Table A1:** Absorption spectrum of the BBH from 0.1 to 10 μg/mL^−1^.

Conc. (mmol/L)	OD Value Absorbance Value	Absorbance Value
0.1	3.1123	0.0537
0.2	3.1581	0.0995
0.4	3.2017	0.1431
0.6	3.2441	0.1735
0.8	3.2725	0.2139
1.0	3.3204	0.2618
10	6.0000	2.9414

**Table A2:** The circle of inhibition of the samples for twenty-four hours.

Samples	*E. coli* Inhibition Zone (mm)	*S. aureus* Inhibition Zone (mm)	*C. albicans* Inhibition Zone (mm)
BBH	20.3 ± 0.5	18.3 ± 1.5	18.3 ± 0.5
CS/CMCD/BBH	10.6 ± 0.1	15.3 ± 0.5	12.6 ± 0.5
CS	5.3 ± 0.5	8 ± 0.1	6.6 ± 0.5
CS/CMCD	9.6 ± 0.5	8 ± 0.1	5.6 ± 0.5

**Table A3:** The circle of inhibition of the samples for forty-eight hours.

Samples	*E. coli* Inhibition Zone (mm)	*S. aureus* Inhibition Zone (mm)	*C. albicans* Inhibition Zone (mm)
BBH	21.3 ± 0.5	19.3 ± 0.5	18.3 ± 0.5
CS/CMCD/BBH	11.6 ± 1.1	16.3 ± 0.05	15 ± 0.5
CS	6.3 ± 0.5	8.6 ± 0.1	7 ± 0.1
CS/CMCD	10.3 ± 1.1	8.6 ± 0.1	8 ± 0.1

**Table A4:** The sample inhibition zone for seventy-two hours.

Samples	*E. coli* Inhibition Zone (mm)	*S. aureus* Inhibition Zone (mm)	*C. albicans* Inhibition Zone (mm)
BBH	22.3 ± 0.5	20 ± 1	19.3 ± 0.5
CS/CMCD/BBH	12.1 ± 0.2	16 ± 0.5	16.3 ± 0.5
CS	7.6 ± 0.1	9.3 ± 0.5	8 ± 0.1
CS/CMCD	10 ± 0.1	9.3 ± 0.5	8.3 ± 0.5
